# Long-Term Survival Impact of High-Grade Complications after Liver Resection for Hepatocellular Carcinoma: A Retrospective Single-Centre Cohort Study

**DOI:** 10.3390/medicina58040534

**Published:** 2022-04-12

**Authors:** Chin-Wen Kuo, Hsiang-Ling Wu, Chun-Cheng Li, Juan P. Cata, Hsin-Yi Liu, Ming-Chih Hou, Yih-Giun Cherng, Ying-Hsuan Tai

**Affiliations:** 1 Department of Anaesthesiology, Shuang Ho Hospital, Taipei Medical University, New Taipei City 23561, Taiwan; 19152@s.tmu.edu.tw (C.-W.K.); 15193@s.tmu.edu.tw (C.-C.L.); 18384@s.tmu.edu.tw (H.-Y.L.); stainless@s.tmu.edu.tw (Y.-G.C.); 2 Department of Anaesthesiology, School of Medicine, College of Medicine, Taipei Medical University, Taipei 11031, Taiwan; 3 Department of Anaesthesiology, Taipei Veterans General Hospital, Taipei 11217, Taiwan; hlwu9@vghtpe.gov.tw; 4 School of Medicine, National Yang Ming Chiao Tung University, Taipei 11221, Taiwan; mchou@vghtpe.gov.tw; 5 Department of Anaesthesiology and Perioperative Medicine, The University of Texas MD Anderson Cancer Centre, 1515 Holcombe Blvd, Unit 409, Houston, TX 77030, USA; jcata@mdanderson.org; 6 Division of Gastroenterology and Hepatology, Department of Medicine, Taipei Veterans General Hospital, Taipei 11217, Taiwan

**Keywords:** cancer recurrence, hepatectomy, hepatic cancer, mortality, survival

## Abstract

*Background and Objectives*: Although complications after liver resection for hepatic cancer are common, the long-term impact of these complications on oncological outcomes remains unclear. This study aimed to investigate the potential effect of high-grade postoperative complications on long-term mortality and cancer recurrence after surgical resection of hepatocellular carcinoma. *Materials and Methods*: In a retrospective cohort study, patients undergoing curative liver resection for primary hepatocellular carcinoma between 2005 and 2016 were evaluated. The Clavien–Dindo (CD) grading system was used to classify patients into two groups of either high-grade complications (grade III or IV) or none or low-grade complications (grade 0 to II) within 30 days after surgery. The primary endpoint was all-cause mortality. Secondary endpoints were cancer-specific mortality and cancer recurrence. Weighted Cox proportional hazards regression models were used to calculate the adjusted hazard ratio (aHR) with a 95% confidence interval (CI) for the outcomes of interest. *Results*: A total of 1419 patients with a median follow-up time of 46.6 months were analysed. Among them, 93 (6.6%) developed high-grade complications after surgery. The most common complications were bile leakage (*n* = 30) in CD grade III and respiratory failure (*n* = 13) in CD grade IV. High-grade complications were significantly associated with all-cause mortality (aHR: 1.78, 95% CI: 1.55–2.06) and cancer-specific mortality (aHR: 1.34, 95% CI: 1.13–1.60), but not cancer recurrence (aHR: 0.92, 95% CI: 0.84–1.02). Independent influential factors for complications were sex, diabetes mellitus, clinically significant portal hypertension, oesophageal varices, multifocal cancer, intraoperative blood loss, and anaesthesia duration. *Conclusions*: Patients who had high-grade postoperative complications had a greater risk of long-term mortality after liver resection for hepatocellular carcinoma. Prevention of postoperative complications may serve as an effective strategy for improving long-term survival.

## 1. Introduction

Hepatocellular carcinoma (HCC) is the most common type of primary liver cancer and is ranked as the third most common cause of cancer-related death worldwide [1]. Surgical resection of primary tumours remains the mainstay treatment modality for patients with resectable HCC. However, the long-term prognosis after surgical resection remains very poor, with rates of 55.9% and 42.3% reported for 1-year and 2-year overall survival, respectively [2,3]. Despite recent advances in surgical techniques and perioperative care, patients undergoing liver resections for HCC are still at high risk of postoperative complications, with a reported rate of 20% to 50% [4,5,6].

Postoperative complication is an established risk factor for short-term adverse events after liver resection, including mortality, readmission, reoperation, prolonged length of hospital stay, and greater medical expenditures [7,8,9,10,11]. Mounting evidence indicates that postoperative complications may also affect long-term survival among patients that survive postoperative complications, irrespectively of the preoperative patient characteristics [12,13]. For patients with cancer, postoperative complications have been demonstrated to be associated with greater long-term mortality and cancer recurrence after surgical resection of lung cancer [14], gastric cancer [15], and pancreatic cancer [16].

Considering that some evidence-based interventions have proven effective in reducing postoperative complications [17], it is important to elucidate the potential impact of these complications on long-term prognoses after liver resection for HCC. This will justify and encourage future studies to examine the potential survival benefits of prophylactic measures for postoperative complications [17]. However, at present, there is no agreement as to whether postoperative complications adversely affect long-term oncological outcomes in HCC, with greater risks reported in some studies [18,19,20,21,22,23,24] but not in others [25,26]. Major limitations of previous studies have been small numbers of subjects (<1000) [18,19,20,21,22,24,25,26], absence of standard definitions for postoperative complications (e.g., the Clavien–Dindo (CD) grading system) [18,19,21], and no evaluation of cancer-specific mortality [18,19,20,21,22,23,24,25,26] or cancer recurrence [19]. In addition, some previous studies included surgeries that were performed before 2010, which made it difficult to reflect recent refinements in surgical techniques and cancer treatment [18,19,20,21,25]. Overall, current evidence is inadequate and not conclusive enough to confirm or refute the long-term survival impact of postoperative complications in patients with HCC.

This study aimed to evaluate the putative effect of high-grade postoperative complications on long-term oncological outcomes after surgical resection for HCC using the CD classification system [27]. Based on current evidence [28], we hypothesised that high-grade postoperative complications were linked to greater all-cause mortality, cancer-specific mortality, and cancer recurrence in patients with HCC. 

## 2. Materials and Methods

### 2.1. Criteria of Patient Selection

**Figure 1:**
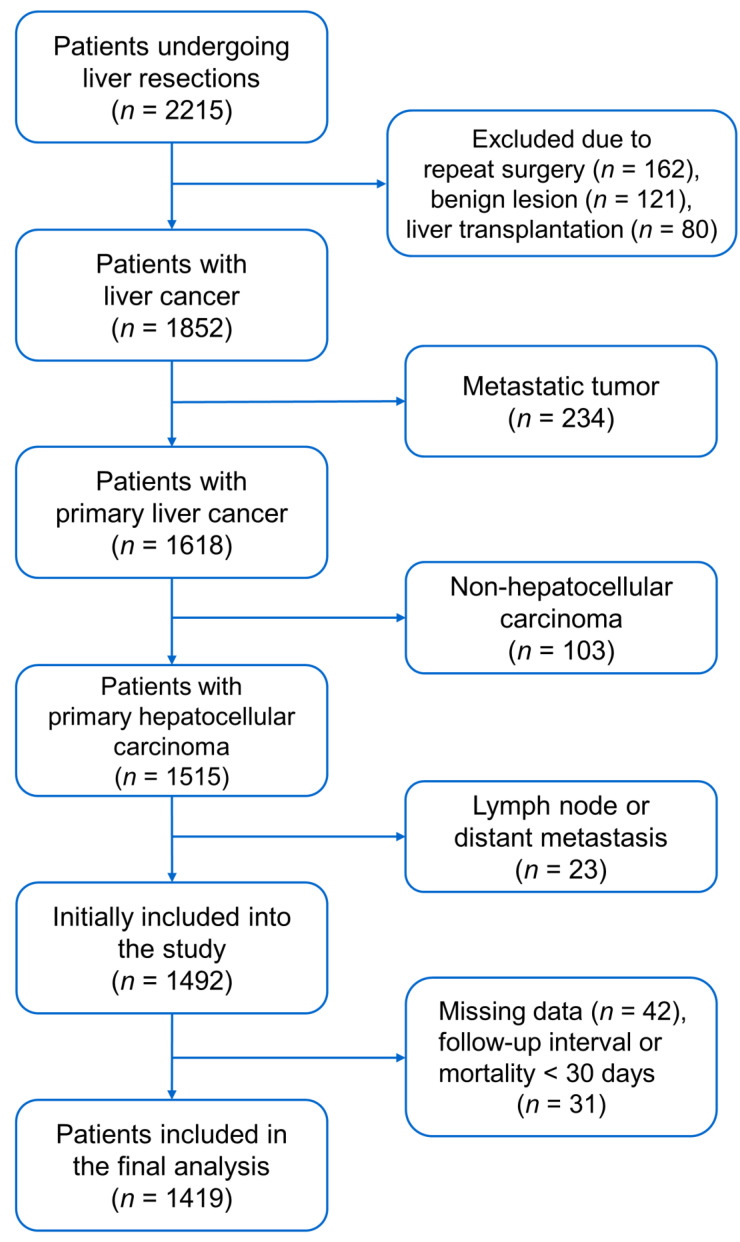
Flow diagram for patient selection.

We obtained approval from the Institutional Review Board of Taipei Veterans General Hospital in Taiwan (IRB-TPEVGH, No. 2021-07-035BC). Written informed consent was waived by the Institutional Review Board, and all the study methods were conducted in accordance with the institutional ethical standards of the responsible committee on human experimentation. We reviewed the medical records of 2215 patients consecutively undergoing hepatic resections at Taipei Veterans General Hospital between 2005 and 2016. Patients were excluded for the following conditions: repeat surgery, liver transplantation, pathology-proven benign lesion, metastatic cancer, non-HCC cancer, lymph node or distant metastasis, missing data, and follow-up interval or mortality < 30 days after surgery. A total of 1419 patients were included for analyses (Figure 1). 

### 2.2. Postoperative Complications

Complications within a 30-day postoperative period were recorded and ranked using the CD classification, a representative grading system for postoperative complications [27]. This study sought to investigate the association between high-grade complications, defined as a CD grade III or IV (grade III: requiring surgical, endoscopic, or radiological interventions; grade IV: requiring intensive care management), and HCC outcomes, because such patients need invasive interventions [27]. In case of multiple complications, the highest grade was retained for analyses. Patients were divided into two groups of either high-grade complication (CD grade III or IV) or none or low-grade complications (CD grade 0 to II, as controls).

### 2.3. Primary and Secondary Endpoints

The primary endpoint was all-cause mortality. Secondary endpoints were cancer-specific mortality and cancer recurrence. Survival time was defined as the interval between the date of the operation and the date of death or recurrence. For patients without death or recurrence, survival times were the corresponding censored observation. Patients were followed up to 30 September 2018. 

### 2.4. Covariates for Adjustment

The institutional electronic medical database was utilised to collect factors potentially associated with oncological outcomes after surgical resection of HCC. The clinical covariates included American Society of Anaesthesiologists (ASA) physical status, viral serology [29], liver cirrhosis and Child–Pugh class [30], clinically significant portal hypertension (hepatic venous pressure gradient ≥ 10 mm Hg), presence of oesophageal varices, and coexisting diseases (alcoholism, diabetes mellitus, and chronic kidney disease) (Table 1). Preoperative records of radiofrequency ablation, trans-arterial chemoembolization, and percutaneous ethanol injection were also considered for analyses. Preoperative laboratory tests, including serum aspartate aminotransferase (AST), alanine aminotransferase (ALT), total bilirubin, alpha-fetoprotein, and albumin, were also considered [30,31]. Surgical characteristics were extent of hepatectomy (>2 Couinaud liver segments or not), use of laparoscopic or robotic techniques, surgical margin, epidural blockade [32,33], intraoperative blood loss and transfusion (red blood cells, fresh frozen plasma or platelets) [34,35], and anaesthesia duration. Pathology features were tumour size and number, cell differentiation, microvascular invasion, and extracapsular invasion [36,37]. Patients were classified according to the Barcelona Clinic Liver Cancer (BCLC) staging system [38]. The data were collected by a specialist anaesthesiologist not involved in the statistical analysis. The authors verified the quality of data through random sampling.

### 2.5. Surgical Techniques and Cancer Surveillance

At the medical centre, all liver resections were performed by experienced general surgeons. The liver parenchymal transection was performed using a clamp-crushing method, and the Pringle’s manoeuvre and argon beam coagulator were used to halt haemorrhage. Laparoscopic or robotic surgery was used in selected patients from July 2011. All surgeries included in this study were performed for curative intent.

For postoperative surveillance, patients received routine ultrasonography, computed tomography, or magnetic resonance imaging every four months for two to three years, then every six months. In addition, the serum concentration of alpha-fetoprotein was checked every four months for two to three years, then every six months. Bone scintigraphy or positron emission tomography were used for suspected locoregional recurrence or distant metastases. If the diagnosis was equivocal, a biopsy was used to confirm the presence of recurrent disease.

### 2.6. Sample Size Estimation

According to previous studies, at least 1175 patients are needed to detect a hazard ratio (HR) of 1.39 for all-cause mortality, accepting a type I error of 5% and a type II error of 20%, with an incidence of high-grade complications of 6.6% in this study [28,39]. We included 1419 patients in the analysis, which met the minimal requirements for sample size.

### 2.7. Statistical Analysis

For baseline patient characteristics, Shapiro–Wilk tests were used as normality tests. Normally distributed variables were presented as the mean ± standard deviation. Non-normally distributed data were presented as the median with interquartile range. Logarithmic transformation was performed to reduce skewness of non-normal continuous variables, including intraoperative blood loss and anaesthesia duration. The distribution of patient characteristics was compared between the high-grade complication group and the control group using either independent *t* tests or Mann–Whitney U tests for continuous variables and chi-square tests or Fisher’s exact tests for categorical variables, as appropriate. Kaplan–Meier curves and log-rank tests were used to compare the cumulative incidences of all-cause mortality, cancer-specific mortality, and cancer recurrence between groups. Univariate Cox proportional hazard regression analysis was conducted to examine the effects of high-grade complications and other covariates on oncological outcomes. 

We used inverse probability treatment weighting (IPTW) as the primary statistical approach because IPTW decreases inherent bias in patient and disease attributes that can affect whether patients do or do not have an exposure factor [40]. IPTW adjusts for the confounding effects that individual covariates could exert on long-term cancer outcomes, whilst retaining the patient sample and statistical power [40]. The IPTW analysis was performed as follows [40]. First, binary logistic regression analysis was used to estimate the probability of developing a high-grade complication based on a list of patient characteristics (Supplementary Table S1). The inverse of the estimated probability was then used for weighted Cox regression analyses, and 1% of subjects at the end of the weighting distribution were truncated to reduce the effect of large weights on study results. The weighted Cox regression model was implemented to assess the independent effects of high-grade postoperative complications on the outcomes of interest. Additionally, multivariable logistic regression analysis was performed using a backward variable elimination process with the entry and removal significance criteria of 0.1 and 0.05, respectively, to identify the influential factors of high-grade complications. We considered *p* < 0.05 to be statistically significant for a two-sided test. All statistical analyses were conducted using SAS software, version 9.4 (SAS Institute Inc., Cary, NC, USA). 

## 3. Results

### 3.1. Patient Characteristics

A total of 1419 patients had a median follow-up time of 46.6 months, with an interquartile range of 22.6 to 80.3 months. Among them, 93 (6.6%) patients developed high-grade complications (CD grade III: 72 and grade IV: 21). Table 1 shows the patient, clinical, and pathological characteristics of the included subjects. Compared with patients with none or low-grade postoperative events, those with high-grade complications were more likely to be older and to have diabetes mellitus, ASA class ≥ 3, a higher level of serum AST, and a lower level of serum albumin. CD grade III or IV complications were also more common in patients with multifocal cancer, >2 segments of liver resected, greater intraoperative blood loss, and a higher transfusion rate. The anaesthesia duration was longer in those with a high-grade complication. The most common complications were bile leakage in CD grade III and respiratory failure in CD grade IV (Table 2).

### 3.2. All-Cause Mortality

**Figure 2:**
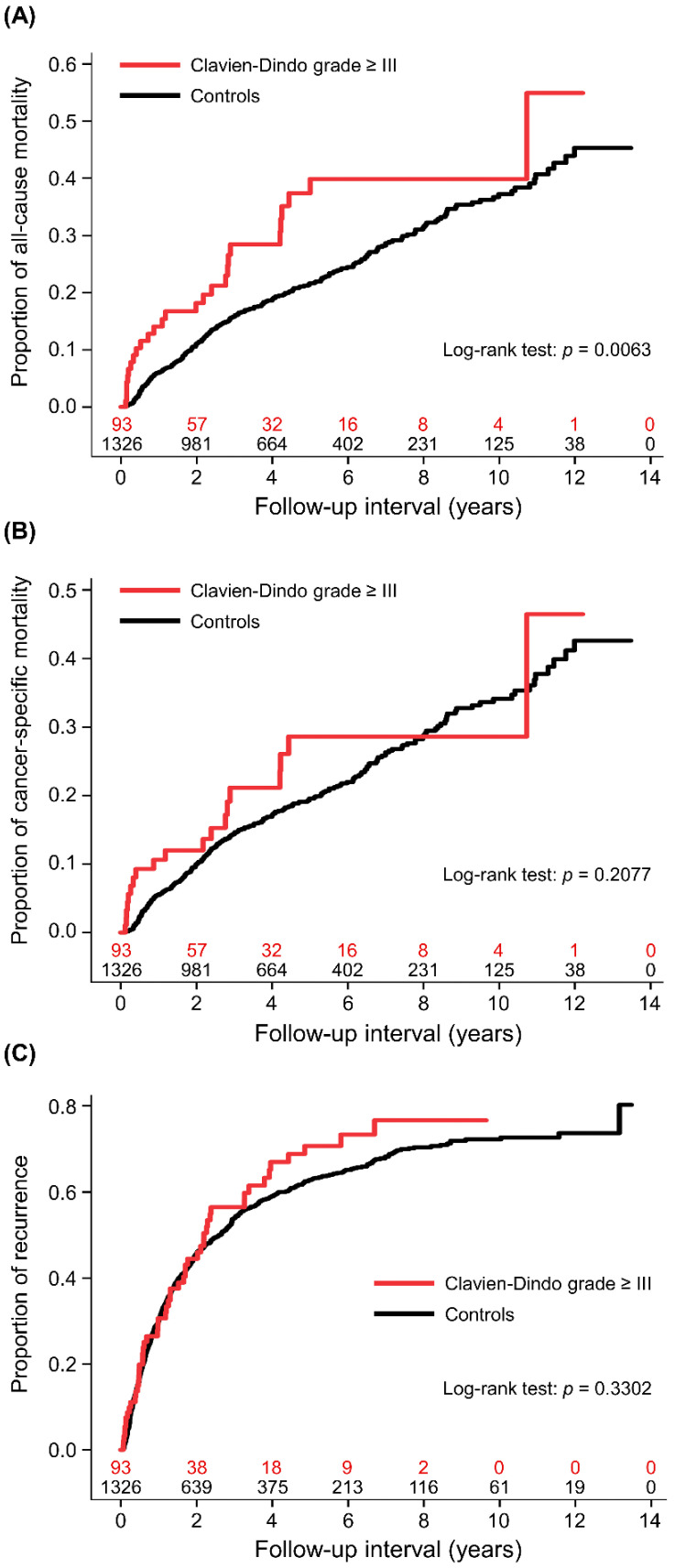
Cumulative incidences of patients with high-grade complications and controls. (**A**) All-cause mortality, (**B**) cancer-specific mortality, and (**C**) cancer recurrence.

The 1-, 3-, and 5-year cumulative all-cause mortality rates were 6.0% (95% CI (confidence interval): 4.6–7.4), 15.9% (95% CI: 13.7–18.1), and 21.6% (95% CI: 19.1–24.1), respectively, in the controls, and 14.1% (95% CI: 6.7–21.5), 28.4% (95% CI: 17.8–39.0), and 39.9% (95% CI: 27.0–52.8), respectively, in those with high-grade complications. In the univariate analysis, postoperative complications were associated with a higher risk of all-cause mortality (crude HR: 1.72 (95% CI: 1.16–2.55, *p* = 0.0069; Figure 2A)). The remaining variables associated with all-cause mortality were age, diabetes mellitus, chronic kidney disease, liver cirrhosis, higher Child–Pugh class, clinically significant portal hypertension, oesophageal varices, preoperative levels of haemoglobin, international normalised ratio, total bilirubin, AST, alpha-fetoprotein, and albumin. Patients had greater all-cause mortality if they had an advanced BCLC stage, larger tumours, multifocal cancer, poor or undifferentiated histology, pathological microvascular invasion, extracapsular invasion, and a positive surgical margin. Other variables were preoperative trans-arterial chemoembolization, radiofrequency ablation or percutaneous ethanol injection, >2 segments of hepatectomy, intraoperative blood loss and transfusion, anaesthesia duration, and operation period (Table 3). The weighted Cox regression models showed that patients with a high-grade complication had a significantly higher risk of all-cause mortality (adjusted HR: 1.78 (95% CI: 1.55–2.06, *p* < 0.0001)).

### 3.3. Cancer-Specific Mortality

The 1-, 3-, and 5-year cumulative cancer-specific mortality rates were 5.4% (95% CI: 4.2–6.6), 14.4% (95% CI: 12.4–16.4), and 19.6% (95% CI: 17.1–22.1), respectively, in the controls, and 10.6% (95% CI: 3.9–17.3), 21.1% (95% CI: 11.3–30.9), and 28.6% (95% CI: 16.6–40.6), respectively, in those with high-grade complications. In the univariate analysis, the association between complications and cancer-specific mortality was non-significant (crude HR: 1.35 (95% CI: 0.85–2.15, *p* = 0.2081; Figure 2B)). There were some common variables which were also associated with all-cause mortality. The unique association for greater cancer-specific mortality was preoperative ALT level (Table 3). After adjusting for covariates in the weighted Cox regression model, high-grade complications were significantly associated with cancer-specific mortality (adjusted HR: 1.34 (95% CI: 1.13–1.60, *p* = 0.0009)). 

### 3.4. Cancer Recurrence

The 1-, 3-, and 5-year cumulative recurrence rates were 30.1% (95% CI: 27.6–32.6), 53.9% (95% CI: 51.2–56.6), and 62.8% (95% CI: 59.9–65.7), respectively, in the controls, and 30.6% (95% CI: 20.4–40.8), 56.5% (95% CI: 45.1–67.9), and 70.7% (95% CI: 59.5–81.9), respectively, in those with high-grade complications. The univariate analysis showed that the association between complications and cancer recurrence was non-significant (crude HR: 1.15 (95% CI: 0.87–1.52, *p* = 0.3307; Figure 2C)). The variables associated with cancer recurrence are shown in Table 3. In the weighted Cox regression model, the association between complications and cancer recurrence remained non-significant (adjusted HR: 0.92 (95% CI: 0.84–1.02, *p* = 0.0973)).

### 3.5. Influential Factors for High-Grade Complications

Preoperative influential factors for high-grade complications included sex (male vs. female, odds ratio (OR): 0.48, 95% CI: 0.28–0.84, *p* = 0.0102), diabetes mellitus (OR: 2.76, 95% CI: 1.66–4.57, *p* = 0.0001), clinically significant portal hypertension (OR: 0.25, 95% CI: 0.08–0.79, *p* = 0.0177), oesophageal varices (OR: 3.19, 95% CI: 1.26–8.09, *p* = 0.0144), and multifocal cancer (OR: 2.18, 95% CI: 1.30–3.67, *p* = 0.0033). Intraoperative influential factors were intraoperative blood loss (OR: 1.61, 95% CI: 1.29–2.02, *p* < 0.0001, on base-2 logarithmic scale) and anaesthesia duration (OR: 2.35, 95% CI: 1.25–4.40, *p* = 0.0077, on base-2 logarithmic scale) (Table 4).

## 4. Discussion

In this study, we found that high-grade postoperative complications were significantly associated with greater all-cause mortality and cancer-specific mortality after liver resection for HCC, but cancer recurrence was not affected. Our analyses identified several influential factors for postoperative complications, including sex, diabetes mellitus, Child–Pugh class, multifocal cancer, intraoperative blood loss, and anaesthesia duration. We used a large single-centre cohort and adjusted for a detailed list of patient and disease characteristics to investigate the putative impact of postoperative complications on long-term oncological outcomes after surgical resection of HCC. This evidence supports the importance of preventing postoperative complications as a practicable strategy of improving long-term survival in patients following liver resection for HCC.

Our results suggest that high-grade complications after hepatic resections might increase the risk of all-cause mortality in patients with HCC, in agreement with some previous studies [18,19,20,21,22,23,24] but not others [25,26]. The present study also demonstrated no definite association between postoperative complications and cancer recurrence, in line with some studies [18,20,25,26] but contrasting with others [21,22,23,24]. Differences in the types and severity of complications are potentially responsible for these inconsistent findings. Two Asian studies recently reported a significant association between postoperative infectious complications and cancer recurrence after surgical resection of HCC [23,24]. Host immunity may underlie the increased recurrence among patients who developed infectious complications [23,24]. Compared with these two studies [23,24], most postoperative complications in our study were non-infectious. Regarding the severity of complications according to the CD classification, previous studies demonstrated a significant association between complication severity and increased long-term mortality rather than cancer recurrence [20,22]. Noticeably, few studies have evaluated the severity threshold for when postoperative complications exert a long-term survival impact. More studies are needed to elucidate the prognostic role of postoperative complications with varying severity after cancer surgery.

The biological mechanism underlying greater long-term mortality in patients who develop postoperative complications remains unclear. Prior studies have shown that greater intraoperative blood loss and perioperative transfusion of allogeneic blood were potentially linked to compromised immune function and adversely affected long-term survival and cancer control [34,35,41]. Additionally, it is uncertain whether the association between postoperative complications and cancer outcomes is causative or simply correlative. Our results showed that patients with pre-existing diabetes mellitus and a higher Child–Pugh class were susceptible to severe postoperative complications, and both factors are established prognostic factors for oncological outcomes in HCC [30,42]. Postoperative complications could be a proxy of worse patient health, more aggressive tumours, and more extensive surgical resections, which can increase long-term mortality, instead of being a direct cause of mortality by themselves.

The present findings justify the prevention of postoperative complications as a method of improving long-term survival after liver resection of HCC. Our analyses identified some modifiable factors for postoperative complications, including diabetes mellitus, intraoperative blood loss, and anaesthesia duration. First, Hosokawa et al. have previously indicated that diabetic patients with inadequate glycaemic control were at higher risk of tumour recurrence and early mortality after radiofrequency ablation therapy for HCC [43]. Accordingly, close monitoring and optimal control of perioperative blood glucose levels are pivotal in the prevention of postoperative complications and the improvement of the patient’s oncological prognosis. Second, since intraoperative haemorrhage and allogeneic blood transfusions may exert a detrimental effect on long-term survival after cancer surgery, strategies aimed at minimizing surgical bleeding should be further developed, particularly for patients with cirrhotic liver [34,35,41]. Third, implementation of enhanced recovery after surgery protocol has been proven safe and effective in decreasing postoperative complications and improving short-term and long-term survival after cancer surgery [44,45,46]. Reduced operative duration should be regarded as a universal goal for surgeons [47]. Further studies are warranted to develop and assess measures which can shorten operative times.

There were some limitations to the present study. First, this study was retrospective, and therefore unrecorded variables (i.e., intraoperative blood pressure, perioperative myocardial injury, frailty, and sarcopenia) could not be further analysed and controlled. Second, we did not analyse low-grade complications. Consequently, our results were not generalizable to these patient populations. Third, the number of high-grade complications in our study was relatively small, which might produce underpowered statistics in the analyses. Fourth, our medical database did not contain the information about the eligibility and receipt of liver transplantation after liver resection for HCC, which might confound the long-term survival in our cohort. Fifth, the sero-epidemiology of hepatitis virus infection and the coverage of hepatitis B vaccination in Taiwan are different from western countries, which might limit the generalisability of our findings [48]. Last, we only included intraoperative blood transfusions in the analysis. Therefore, the impact of postoperative blood product administration on complications and survival remains unknown in our patient population. 

## 5. Conclusions

Patients who developed high-grade postoperative complications had greater long-term risks of all-cause mortality and cancer-specific mortality after liver resection for HCC. Female, diabetes mellitus, higher Child–Pugh class, multifocal tumour, greater intraoperative blood loss, and longer anaesthesia duration were risk factors for high-grade complications after surgical resection of HCC. These findings highlight the importance of preventing postoperative complications as a clinical strategy for improving long-term survival after liver resection for HCC. Adequate glycaemic control, optimal surgical haemostasis, and reduced operation duration may play an essential role in decreasing complications and early mortality after liver resection. Future studies are warranted to evaluate the clinical benefits of prophylactic measures for postoperative complications in long-term cancer control and patient survival.

## Figures and Tables

**Table 1:** Patient demographics, and clinical and pathological characteristics.

	CD Grade ≥ III (*n* = 93)	CD Grade (0–II) (*n* = 1326)	*p*
Age, year	63.7 ± 12.2	61.0 ± 12.9	0.0488
Sex, male	66 (71.0%)	1018 (76.8%)	0.2026
ASA class ≥ 3	42 (45.2%)	378 (28.5%)	0.0007
Aetiology of HCC			
HBsAg-positive	58 (62.4%)	894 (67.4%)	0.3159
Anti-HCV Ab-positive	20 (21.5%)	289 (21.8%)	0.9479
Alcoholism	8 (8.6%)	94 (7.1%)	0.5850
Liver cirrhosis	45 (48.4%)	573 (43.2%)	0.3306
Child–Pugh classification			0.5851
Class A	41 (44.1%)	530 (40.0%)	
Class B	4 (4.3%)	43 (3.2%)	
Clinically significant portal hypertension	9 (9.7%)	129 (9.7%)	0.9872
Oesophageal varices	9 (9.7%)	74 (5.6%)	0.1036
Diabetes mellitus	42 (45.2%)	298 (22.5%)	<0.0001
Chronic kidney disease	11 (11.8%)	124 (9.4%)	0.4314
Preoperative laboratory tests			
Haemoglobin, g·dL^−1^	13.1 ± 1.7	13.4 ± 1.7	0.1929
Platelet count, 10^3^·μL^−1^	176.8 ± 78.9	179.5 ± 78.4	0.7501
Thrombocytopenia	34 (36.6%)	531 (40.1%)	0.5068
International normalised ratio	1.05 ± 0.07	1.07 ± 0.82	0.3066
Total bilirubin ≥ 1.0 mg·dL^−1^	24 (25.8%)	298 (22.5%)	0.4630
AST > 40 IU·L^−1^	57 (61.3%)	593 (44.9%)	0.0022
ALT > 40 IU·L^−1^	51 (54.8%)	623 (47.0%)	0.1425
Alpha-fetoprotein > 20 ng·mL^−1^	44 (48.9%)	655 (50.8%)	0.7239
Albumin ≤ 3.5 g·dL^−1^	12 (13.0%)	95 (7.2%)	0.0421
Serum creatinine, mg·dL^−1^	0.99 ± 0.72	1.02 ± 0.83	0.7167
BCLC stage			0.0284
Stage 0	8 (8.6%)	180 (13.6%)	
Stage A	33 (35.5%)	589 (44.4%)	
Stage B	52 (55.9%)	557 (42.0%)	
Pathologic characteristics			
Tumour diameter > 5 cm	38 (40.9%)	479 (36.1%)	0.3589
Multifocal cancer	36 (38.7%)	273 (20.6%)	<0.0001
Poor or undifferentiated histology	30 (32.3%)	468 (35.3%)	0.7674
Microvascular invasion	65 (69.9%)	906 (68.3%)	0.7534
Extracapsular invasion	33 (35.5%)	563 (42.5%)	0.1877
Positive surgical margin	10 (10.8%)	78 (5.9%)	0.0598
Preoperative TACE/RFA/PEI	9 (9.7%)	117 (8.8%)	0.7796
Surgical and anaesthetic management			
Hepatectomy > 2 segments	49 (52.7%)	463 (34.9%)	0.0006
Laparoscopic or robotic surgery	4 (4.3%)	83 (6.3%)	0.4467
Epidural analgesia	35 (37.6%)	509 (38.4%)	0.8854
Intraoperative blood loss, mL	1050 (600–2100)	600 (300–1150)	<0.0001
Blood transfusion rate	81 (87.1%)	799 (60.3%)	<0.0001
Anaesthesia duration, min	420 (315–540)	335 (285–420)	<0.0001
Operation period (2011–2016)	54 (58.1%)	691 (52.1%)	0.2664

Values were mean ± SD, median (interquartile range), or counts (percent). ALT: alanine aminotransferase; Anti-HCV Ab: hepatitis C antibody; ASA: American Society of Anaesthesiologists; AST: aspartate aminotransferase; BCLC: Barcelona Clinic Liver Cancer; CD: Clavien–Dindo classification; HBsAg: hepatitis B surface antigen; HCC: hepatocellular carcinoma; PEI: percutaneous ethanol injection; RFA: radiofrequency ablation; TACE: trans-arterial chemoembolization.

**Table 2:** Clavien–Dindo grade III and IV complications within 30 days after liver resection.

Clavien–Dindo Grade III	Count (Percentage) ^†^
Bile leakage	30 (35%)
Pleural effusion	16 (19%)
Wound complication	12 (14%)
Intra-abdominal abscess	8 (9%)
Massive ascites	4 (5%)
Hepatic haemorrhage	4 (5%)
Obstructive jaundice	2 (2%)
Liver abscess	2 (2%)
Pneumothorax	2 (2%)
Occlusion of common hepatic duct	1 (1%)
Oedematous change of bile duct wall	1 (1%)
Peritonitis	1 (1%)
Duodenal ulcer bleeding	1 (1%)
Intestinal obstruction	1 (1%)
Acute kidney injury	1 (1%)
Clavien–Dindo Grade IV	Count (Percentage)
Respiratory failure	13 (62%)
Cerebral infarction	2 (10%)
Sepsis	2 (10%)
Multiorgan failure	2 (10%)
Hepatic failure	1 (5%)
Myocardial infarction	1 (5%)

^†^ 14 patients had 2 complications.

**Table 3:** Univariate analysis of mortality and cancer recurrence.

	All-Cause Mortality		Cancer-Specific Mortality		Cancer Recurrence	
	cHR (95% CI)	*p*	cHR (95% CI)	*p*	cHR (95% CI)	*p*
Clavien–Dindo grade ≥ III vs. controls	1.72 (1.16–2.55)	0.0069	1.35 (0.85–2.15)	0.2081	1.15 (0.87–1.52)	0.3307
Age, year	1.01 (1.00–1.02)	0.0392	1.01 (1.00–1.02)	0.1303	1.00 (1.00–1.01)	0.2228
Sex, male	1.04 (0.80–1.36)	0.7479	1.02 (0.77–1.34)	0.9093	1.08 (0.92–1.27)	0.3644
ASA class ≥ 3	1.24 (0.98–1.57)	0.0737	1.18 (0.92–1.52)	0.2016	1.07 (0.92–1.24)	0.3653
HBsAg-positive	1.00 (0.79–1.26)	0.9694	1.03 (0.80–1.32)	0.8221	1.10 (0.95–1.28)	0.1866
Anti-HCV Ab-positive	1.09 (0.84–1.42)	0.4963	1.02 (0.77–1.35)	0.8773	1.16 (0.99–1.36)	0.0625
Alcoholism	0.79 (0.49–1.26)	0.3184	0.84 (0.52–1.38)	0.4970	0.88 (0.68–1.15)	0.3593
Liver cirrhosis	1.62 (1.30–2.01)	<0.0001	1.63 (1.29–2.05)	<0.0001	1.38 (1.21–1.58)	<0.0001
Child–Pugh class B	2.32 (1.53–3.52)	<0.0001	2.29 (1.47–3.57)	0.0003	1.74 (1.25–2.43)	0.0009
Clinically significant portal hypertension	1.85 (1.37–2.52)	<0.0001	1.90 (1.38–2.62)	<0.0001	1.56 (1.26–1.93)	<0.0001
Oesophageal varices	2.31 (1.61–3.31)	<0.0001	2.39 (1.64–3.49)	<0.0001	1.53 (1.17–2.02)	0.0022
Diabetes mellitus	1.42 (1.12–1.81)	0.0040	1.35 (1.04–1.75)	0.0226	1.11 (0.95–1.29)	0.1988
Chronic kidney disease	1.64 (1.20–2.23)	0.0018	1.43 (1.02–2.02)	0.0410	0.95 (0.75–1.20)	0.6496
Haemoglobin, g·dL^−1^	0.88 (0.83–0.94)	<0.0001	0.89 (0.84–0.96)	0.0009	0.96 (0.93–1.00)	0.0470
Platelet count, 10^3^·μL^−1^	1.00 (1.00–1.00)	0.8567	1.00 (1.00–1.00)	0.7302	1.00 (1.00–1.00)	0.8497
Thrombocytopenia	1.15 (0.92–1.44)	0.2092	1.14 (0.90–1.44)	0.2878	1.13 (0.99–1.30)	0.0790
International normalised ratio	1.09 (1.02–1.16)	0.0103	1.09 (1.01–1.17)	0.0265	1.04 (0.98–1.11)	0.2155
Total bilirubin ≥ 1.0 mg·dL^−1^	1.36 (1.06–1.73)	0.0141	1.44 (1.11–1.85)	0.0056	1.09 (0.93–1.28)	0.3000
AST > 40 IU·L^−1^	1.98 (1.59–2.47)	<0.0001	2.05 (1.62–2.59)	<0.0001	1.71 (1.50–1.96)	<0.0001
ALT > 40 IU·L^−1^	1.19 (0.96–1.48)	0.1137	1.26 (1.00–1.59)	0.0482	1.24 (1.09–1.42)	0.0015
Alpha-fetoprotein > 20 ng·mL^−1^	1.62 (1.29–2.02)	<0.0001	1.75 (1.38–2.23)	<0.0001	1.63 (1.42–1.86)	<0.0001
Albumin ≤ 3.5 g·dL^−1^	1.74 (1.19–2.54)	0.0040	1.65 (1.09–2.49)	0.0180	1.48 (1.16–1.88)	0.0013
Serum creatinine, mg·dL^−1^	1.08 (0.96–1.21)	0.2076	1.01 (0.85–1.19)	0.9380	0.98 (0.89–1.07)	0.6319
BCLC stage		<0.0001		<0.0001		<0.0001
Stage A vs. 0	1.27 (0.85–1.90)	0.2529	1.40 (0.90–2.19)	0.1410	1.28 (1.01–1.61)	0.0382
Stage B vs. 0	2.57 (1.75–3.79)	<0.0001	2.89 (1.88–4.44)	<0.0001	2.03 (1.62–2.54)	<0.0001
Tumour diameter > 5 cm	2.13 (1.72–2.65)	<0.0001	2.18 (1.73–2.75)	<0.0001	1.56 (1.36–1.79)	<0.0001
Multifocal cancer	1.57 (1.23–2.00)	0.0003	1.58 (1.22–2.04)	0.0006	1.80 (1.55–2.09)	<0.0001
Poor or undifferentiated histology	1.37 (1.10–1.72)	0.0057	1.46 (1.15–1.85)	0.0018	1.43 (1.25–1.64)	<0.0001
Microvascular invasion	1.96 (1.52–2.52)	<0.0001	2.24 (1.70–2.97)	<0.0001	1.71 (1.47–1.99)	<0.0001
Extracapsular invasion	1.36 (1.09–1.69)	0.0057	1.39 (1.10–1.75)	0.0054	1.29 (1.13–1.48)	0.0002
Positive surgical margin	2.79 (1.96–3.99)	<0.0001	2.80 (1.92–4.09)	<0.0001	2.13 (1.65–2.76)	<0.0001
Preoperative TACE/RFA/PEI	1.49 (1.06–2.10)	0.0222	1.61 (1.13–2.30)	0.0080	1.23 (0.98–1.54)	0.0806
Hepatectomy > 2 segments	1.51 (1.22–1.88)	0.0002	1.55 (1.23–1.95)	0.0002	1.27 (1.11–1.46)	0.0007
Laparoscopic or robotic surgery	0.58 (0.30–1.13)	0.1082	0.66 (0.34–1.29)	0.2261	0.77 (0.57–1.06)	0.1098
Epidural blockade	1.05 (0.85–1.31)	0.6367	1.04 (0.82–1.31)	0.7487	1.04 (0.91–1.20)	0.5363
Intraoperative blood loss, mL ^†^	1.35 (1.24–1.47)	<0.0001	1.36 (1.24–1.49)	<0.0001	1.23 (1.17–1.30)	<0.0001
Blood transfusion rate	2.45 (1.90–3.17)	<0.0001	2.68 (2.02–3.54)	<0.0001	1.64 (1.42–1.90)	<0.0001
Anaesthesia duration, min ^†^	1.94 (1.51–2.49)	<0.0001	1.97 (1.51–2.57)	<0.0001	1.59 (1.36–1.85)	<0.0001
Operation period (2011–2016 vs. 2005–2010)	0.69 (0.54–0.88)	0.0024	0.70 (0.54–0.90)	0.0059	0.82 (0.72–0.95)	0.0055

ALT: alanine aminotransferase; Anti-HCV Ab: hepatitis C antibody; ASA: American Society of Anaesthesiologists; AST: aspartate aminotransferase; BCLC: Barcelona Clinic Liver Cancer; cHR: crude hazard ratio; CI: confidence interval; HBsAg: hepatitis B surface antigen; HCC: hepatocellular carcinoma; PEI: percutaneous ethanol injection; RFA: radiofrequency ablation; TACE: trans-arterial chemoembolization. ^†^ On base-2 logarithmic scale.

**Table 4:** Independent factors for Clavien–Dindo grade III or IV complications.

	OR	95% CI	*p*
Sex, male vs. female	0.48	0.28–0.84	0.0102
Diabetes mellitus	2.76	1.66–4.57	0.0001
Clinically significant portal hypertension	0.25	0.08–0.79	0.0177
Oesophageal varices	3.19	1.26–8.09	0.0144
Multifocal cancer	2.18	1.30–3.67	0.0033
Intraoperative blood loss, mL ^†^	1.61	1.29–2.02	<0.0001
Anaesthesia duration, min ^†^	2.35	1.25–4.40	0.0077

CI: confidence interval; OR: odds ratio. ^†^ On base-2 logarithmic scale.

## Data Availability

The data presented in this study are available upon request from the corresponding author. The data are not publicly available due to the regulations of the Institutional Review Board.

## Supplementary Materials

The following supporting information can be downloaded at: https://www.mdpi.com/article/10.3390/medicina58040534/s1, Table S1: The result of logistic regression analysis for inverse probability of treatment weighting models.
