# Supplementary material for: Long-Term Survival Impact of High-Grade Complications after Liver Resection for Hepatocellular Carcinoma: A Retrospective Single-Centre Cohort Study
Source: Medicina (Kaunas). 2022 Apr 12;58(4):534. doi: 10.3390/medicina58040534 (PMC9024755; doi:10.3390/medicina58040534)
Supplement: Supplementary file 1 [file medicina-58-00534-s001.zip › Supplementary materials.pdf]

Supplementary Table S1: The result of logistic regression analysis for inverse probability of treatment weighting models

	**OR**	**95% CI**	***p***
Age, year	1.01	0.99 – 1.04	0.3856
Sex, male	0.49	0.26 – 0.93	0.0301
ASA class ≥ 3	1.48	0.84 – 2.60	0.1729
HBsAg positive	0.89	0.47 – 1.69	0.7126
Anti-HCV Ab positive	0.69	0.32 – 1.50	0.3510
Alcoholism	1.54	0.58 – 4.13	0.3883
Liver cirrhosis	1.31	0.72 – 2.38	0.3759
Child-Pugh class B	1.40	0.34 – 5.74	0.6366
Clinically significant portal hypertension	0.24	0.07 – 0.84	0.0249
Oesophageal varices	2.48	0.89 – 6.93	0.0840
Diabetes mellitus	2.96	1.72 – 5.08	0.0001
Chronic kidney disease	0.74	0.28 – 1.91	0.5308
Haemoglobin, $g \cdot dL^{-1}$	1.02	0.87 – 1.20	0.7873
Platelet count, $10^3 \cdot \mu L^{-1}$	1.00	1.00 – 1.00	0.3015
Thrombocytopenia	0.58	0.27 – 1.27	0.1737
International normalized ratio	1.06	0.04 – 29.37	0.9734
Total bilirubin ≥ 1.0 $mg \cdot dL^{-1}$	1.22	0.66 – 2.27	0.5244
AST > 40 $IU \cdot L^{-1}$	1.14	0.58 – 2.23	0.7047
ALT > 40 $IU \cdot L^{-1}$	1.06	0.56 – 2.01	0.8502
Alpha-fetoprotein > 20 $ng \cdot mL^{-1}$	1.00	0.56 – 1.79	0.9924
Albumin ≤ 3.5 $g \cdot dL^{-1}$	1.60	0.73 – 3.53	0.2396
Serum creatinine, $mg \cdot dL^{-1}$	0.88	0.59 – 1.32	0.5434
BCLC stage			0.3121
Stage A vs. 0	1.56	0.50 – 4.86	0.4453
Stage B vs. 0	2.99	0.68 – 13.05	0.1463
Tumour diameter > 5 cm	0.35	0.13 – 0.96	0.0412
Multifocal cancer	1.79	0.92 – 3.49	0.0858
Poor or undifferentiated histology	0.58	0.21 – 1.59	0.2885
Microvascular invasion	0.72	0.36 – 1.42	0.3367
Extracapsular invasion	0.90	0.49 – 1.62	0.7166
Positive surgical margin	1.32	0.51 – 3.40	0.5703
Preoperative TACE/RFA/PEI	0.59	0.23 – 1.56	0.2921
Hepatectomy > 2 segments	1.33	0.75 – 2.38	0.3320
Laparoscopic or robotic surgery	0.55	0.15 – 1.95	0.3532
Epidural blockade	0.92	0.50 – 1.67	0.7740
Intraoperative blood loss, mL†	1.43	1.10 – 1.86	0.0075
Blood transfusion rate	1.66	0.75 – 3.70	0.2120
Anaesthesia duration, min†	2.58	1.26 – 5.26	0.0092
Operation period (2011-2016 vs. 2005-2010)	1.50	0.76 – 2.94	0.2416

ALT: alanine aminotransferase; Anti-HCV Ab: hepatitis C antibody; ASA: American Society of Anesthesiologists; AST: aspartate aminotransferase; CI: confidence interval; BCLC: Barcelona Clinic Liver Cancer; HBsAg: hepatitis B surface antigen; HCC: hepatocellular carcinoma; OR: odds ratio; PEI: percutaneous ethanol injection; RFA: radiofrequency ablation; TACE: transarterial chemoembolization. † On base-2 logarithmic scale
